# A Comparison of Mechanical Parameters Between the Counter Movement Jump and Drop Jump in Biathletes

**DOI:** 10.2478/v10078-012-0064-y

**Published:** 2012-10-23

**Authors:** Henryk Król, Władysław Mynarski

**Affiliations:** 1 Academy of Physical Education in Katowice, Poland, Department of Biomechanics.; 2 Academy of Physical Education in Katowice, Poland, Department of Recreation.

**Keywords:** biathletes, muscular activity, drop jump, vertical jump

## Abstract

The main objective of the study was to determine to what degree higher muscular activity, achieved by increased load in the extension phase (eccentric muscle action) of the vertical jump, affects the efficiency of the vertical jump. Sixteen elite biathletes participated in this investigation. The biathletes performed tests that consisted of five, single “maximal” vertical jumps (counter movement jump – CMJ) and five, single vertical jumps, in which the task was to touch a bar placed over the jumping biathletes (specific task counter movement jump – SCMJ). Then, they performed five, single drop jumps from an elevation of 0.4m (DJ). Ground reaction forces were registered using the KISTLER 9182C force platform. MVJ software was used for signal processing (Król, 1999) and enabling calculations for kinematic and kinetic parameters of the subject’s jump movements (on-line system). The results indicate that only height of the jump (h) and mean power (P_mean_) during the takeoff are statistically significant. Both h and P_mean_ are higher in the DJ. The results of this study may indicate that elite biathletes are well adapted to eccentric work of the lower limbs, thus reaching greater values of power during the drop jump. These neuromuscular adaptive changes may allow for a more dynamic and efficient running technique.

## Introduction

Early research on human muscles by Komi and Bosco (1978), and Komi (1984; 1986) demonstrated that concentric muscle work was increased when preceded by active stretch (eccentric action). This phenomenon is known as the stretch-shortening cycle (SSC) and has been extensively studied by Wilson et al. (1991), Van Ingen Schenau et al. (1997), Bird and Hudson (1998), Elliot et al. (1999), and Cronin et al. (2001).

Two mechanisms are the basis of both, voluntary and involuntary motor processes involved in the stretch-shortening cycle.

The first mechanism (*the neuromuscular model*) is based on the so-called *stretch reflex*, which is also called the *muscle spindle reflex* or *myotatic reflex*, and on the *Golgi tendon organ reflex* (*GTO reflex;*Radliffe and Farentinos, 1999). These reflexes are governed by two receptors: namely, muscle spindles and the Golgi tendon organ. Each reflex has a distinct facilitation and inhibition purpose. Muscle spindles contain several intrafusal fibers that are arranged in parallel with extrafusal fibers. Muscle spindles provide information about muscle length via sensory neurons. Specifically, spindles detect the rate of change in length (velocity), and report the information to the central nervous system (CNS). In response to a rapid muscle stretch, alpha motor neurons are stimulated causing a contraction to the agonist muscle. The Golgi tendon organs (GTOs) are located in the muscle-tendon complex. These GTOs monitor tension. The GTOs provide an inhibitory reflex on motor neurons of the agonist muscle. Activity of the tendon organs may be overridden, however, by voluntary impulses. Under certain circumstances the GTO may provide an excitatory stimulation, therefore their net effect remains in question.

The second mechanism of the stretch-shortening cycle (*the mechanical model*) involves the use of the elastic energy of the muscle-tendon complex. Elastic energy arises from the returning of the muscle-tendon complex to its original shape after the resolution of the mechanical stress that caused the deformation. This mechanism is possible due to the construction of a muscle-tendon complex, which consists of the contractile and noncontractile elements. Contractile elements are contained within the sarcomere of a muscle and consist of actin and myosin. Non contractile elements consist of the series elastic component (SEC), also known as the muscle-tendon unit (Finni et al., 2003), and the parallel elastic component (PEC), which consists of the connective tissues surrounding individual fibers, muscle bundles, and the entire muscle (i.e. endomysium, perimysium, and epimysium). The *mechanical model* states, that muscle force is increased due to the reutilization of elastic energy by the SEC (Finni et al., 2003). In other words, when a muscle is eccentrically loaded (i.e., stretched) and immediately followed by a concentric action, energy will be produced, stored, and ultimately released from the muscle-tendon unit. Force and power output will then be increased. On the other hand, the *neuromuscular model* suggests that preactivation of a muscle or muscle group is partially responsible for the force and power potentiation during the concentric phase of a SSC.

The vast majority of everyday activities and various sport techniques make use of the stretch-shortening cycle (SSC). This cycle includes the time it takes to switch from the eccentric loading phase of the movement to the concentric power production phase. During an eccentric contraction, the muscle elongates during tension. Elongation takes place because the opposing force is greater than the force being generated. A concentric contraction occurs when the muscle shortens during tension. Shortening appears because the force generated by the muscle is greater than the force opposing it. The rapid eccentric phase of muscle contraction stimulates the muscle spindle and the elastic properties of the muscle. A powerful and immediate concentric contraction is thus facilitated. The time between the eccentric phase and the concentric phase is the amortization phase. To recover part of the energy generated during the eccentric phase of movement, the amortization phase must be short (≤ 200 ms; Wilson et al., 1991; Elliot et al., 1999; Siff, 2000; Knudson, 2007). The ability to shorten the amortization phase and also absorb greater forces during the eccentric contraction leads to greater force production in the concentric phase.

Depending on the type of the activity undertaken, the benefits of a well coordinated SSC movement would be a 10–20% increase in muscle strength compared to using strictly concentric muscle work (Knudson, 2007). It has been established that the mechanical benefits of the SSC are, to a large extent, dependent on the resistance encountered during the movement (Cronin et al., 2001).

In sports training, exercises based on the muscle stretch-shortening cycle are named plyometric exercises or, simply, plyometrics. According to Wilk et al. (1993) plyometrics include a quick powerful movement involving a pre-stretch of the muscle, thereby activating the stretch-shortening cycle. Plyometric exercises involve repeated, rapid, eccentric and concentric movements (via jumping and rebounding or swinging and throwing actions) to increase muscle power.

The jumping, hopping, bounding and throwing movements involved in plyometrics have been part of the human motion landscape throughout the ages. However, it was not until the mid-20th century that these activities were more formally applied to athletic performance enhancement. The first reports about the methods and concept of plyometric exercises were provided by the coaches and scientists from the former USSR (Verkhoshansky, 1968; 1969).

Plyometric exercises stimulate changes in the neuromuscular system. These exercises increase inter group muscle coordination, thus the muscle groups involved in a particular activity produce a faster and stronger response to small and rapid changes in muscle length. Systematic use of these exercises most likely increases the eccentric muscular force tolerance, and improves the potential for concentric work (Komi, 1986). According to Komi (1998), greater eccentric force tolerance can be achieved either by enhancing the stretch reflex interaction or by reducing the inhibitory effect of the GTO reflex. At the same time, it seems that the possibility of weakening the GTO reflex while training, is greater than the enhancement of the first reflex (Zatsiorsky, 1995). This adaptation of the nervous system’s symptom (greater stretch load tolerance) is most commonly assessed using the electromyographic method (EMG). For example, during the depth jump (in the landing phase), a high energy load is exerted on the knee joint straightening and ankle joint flexing muscles. In untrained subjects, a decreased muscle activity induced by tendon reflex is observed (protective action) at the moment of foot contact with the ground. Athletes who on a every day basis use jumping movements, show increased muscle activity (Sale, 1992). This greater muscle activity results from the adaptation (mainly at the spinal cord level) of the CNS to sport-specific activities (Sale, 1992). The higher muscle excitation allows for better use of the SSC during the concentric phase (take-off phase) of the jump that immediately follows the landing phase.

From the physiological point of view, one purpose of plyometric training is to increase the excitability of the neurological receptors. This increase will then improve reactivity of the neuromuscular system (Wilk et al., 1993). According to Gambetta (2007), from the training point of view, the goals of plyometric exercises are: to raise explosive power, to learn to better attenuate ground reaction forces regardless of the sport, and to learn to tolerate as well as use greater stretch loads.

A classical example of a plyometric exercise is the vertical drop jump. A considerable amount of biomechanical research has been done concerning drop jumping (Masamoto et al., 2003; McClymont, 2003; Bober et al., 2006; Marcović, 2005; Zatsiorsky and Kraemer, 2006; Litkowycz et al., 2010). Most types of plyometric exercises include jumps or bounds that are performed on a flat, rigid surface or from an elevation of 0.2 to 0.6m (Walsh et al., 2004; Marković, 2007). According to Komi’s research conducted on volleyball players (described by Sale, 1992), it can be confirmed that the CNS adaptation during sport-specific exercise extends to a full range of loads (various heights of the drop jump). Similarly, the results of female gymnasts, in comparison to the control group, were significantly better in the full range of load extensions. The greatest differences were observed in the highest drop jumps (Sale, 1992).

Previous studies regarding the impact of an increased load during the eccentric phase of the vertical jump were related to those sport disciplines in which this form of movement was specific.

In the this work the impact of the increased load was tested in biathletes, were the eccentric component of muscular work has become more significant, after the introduction of the sprints.

In short distance running and Nordic skiing, as in all jumping exercises the stretch-shortening cycle is of great importance.

The aim of this study was to determine to what degree higher muscular activity which is achieved by increased load in the eccentric phase of the vertical jump affects the efficiency of the vertical jump.

## Material and Methods

Eight male and eight female elite biathletes of the Polish Junior National Team participated in this investigation (Table 1). The research project was approved by the Ethics Committee for Scientific Research at the Academy of Physical Education in Katowice, Poland.

All subjects were tested under the same conditions in laboratory settings. The warm-up included 10 minutes of cycling on an Excalibure Sport ergometer (LODE) at a constant cadence of 70 RPM with a work load of 1.5 W/kg, followed by a 10-minute stretching program. The warm-up was followed by 3 counter movement jumps on a force plate. Afterwards, the subjects were instructed how to perform the jumps: “*jump as high and as fast as possible*”.

Then, tests were performed that consisted of five single “maximal” vertical jumps (counter movement jump – CMJ). A modified version of the test, without the use of arm swings, was adopted (Komi and Bosco, 1978). The task for the biathletes was to touch an overhead bar (five single vertical jumps – specific task counter movement jump – SCMJ). According to earlier studies (Król, 2001), it is known that full engagement of subjects is achieved only when a specific task is presented to them. Athletes who practiced for 6 weeks with the bar improved their jump height about 4cm, and those who practiced without the bar - only about 2cm. The subjects also performed five single drop jumps from an elevation of 0.40 m (DJ). Intervals between consecutive jumps were set on the basis of our prior testing experience. The rest interval between single jumps and particular sets of jumps varied from 45 to 60 s. Ground reaction forces were registered using the KISTLER 9182C force platform. MVJ software was used for signal processing (Król, 1999) and enabling calculations of kinematic (height of the jump - *h*) and kinetic parameters (mean power - *P*_mean_ and peak power -*P_peak_* during the take-off phase) of the subject’s jumping movements (on-line system).

### Statistical analysis

Statistical analysis were carried out using software Statistica v. 8.0 (Statsoft, USA). Descriptive statistics (mean, and standard deviation) for all measured mechanical parameters were calculated. The normality distribution of data was checked with the Shapiro-Wilk test. Because data distribution was not normal, the Wilcoxon Matched Pairs Ranks test was used. Additionally, Spearman’s correlation tests were used to assess the relationships between the years of sport experience, and the measures of the plyometric jumps. The level of significance was set at p < 0.05.

## Results

The height of a vertical jump is considered an athlete’s jumping potential. Based on the results of elite athletes from disciplines such as basketball and volleyball (Sale, 1992), the results of the selected competitors, presented in Table 2 and 3, are relatively poor. During our evaluations, the subjects performed a modified jump version with their arms behind their back. Due to the lack of arm swings, the results of the jumps were approximately 5 cm lower.

In similar conditions, elite athletes from 6 different individual sports and 6 different team sports obtained even lower results (Table 4; Kollias et al., 2004). The results were a surprise, especially when compared to our previous results obtained by first league soccer players (Table 4; Król, 2008). Compared to ski jumpers of the Polish National Team (Table 4; Król, 2007), the height of the CMJ was significantly lower, as we expected.

In studies conducted by Vaverka and Gajda (2008) on male university students who were regularly active in sports, higher results (*h_CMJ_= 0.402±0.05m*) were obtained compared to our results. In this case, however, the counter movement jump was performed allowing the arms swing.

Interestingly, in most of the examined cases, slightly higher values of jump heights (*h*) were achieved when subjects performed the drop jump (Table 2 and 3). This is even more visible when analyzing mean power (*P*_mean_) and peak power (*P_peak_*).

The Wilcoxon Matched Pairs Ranks test was performed in order to verify these differences in CMJ, SCMJ and DJ, because data distribution was not normal. The results are presented in Table 5 and 6. In females only one of the six values in the pairs of considered variables was significantly different (p < 0.05). This included the *height of the jump* (*h*) between the CMJ and the DJ. In case of male biathletes the only statistically significant difference was observed in *mean power* (*P*_mean_) between the CMJ and the DJ.

In addition, Table 7 shows the correlation coefficients among the results of the DJ and the CMJ tests and years of sport experience.

Correlation analysis (Table 7) shows that the effect of the drop jump measured by the difference of jump height (*h*) in the DJ and the CMJ is not dependent on sport experience (*R = −0.027, p = 0.811).* However, statistically significant correlations were found between sport experience and the difference between power output during the take-off phase in the DJ and the CMJ (for mean power *R = 0.264, p = 0.019* and for peak power *R = 0.358, p* = *0.001*). In both cases (*mean power* and *peak power*) the effect of the drop jump was dependent on sport experience.

## Discussion

The results given in Table 5 and 6 confirm proper adaptive changes in the neuro-muscular system of biathletes due to an increased workload in the eccentric form of movement. Such a workload refers to the drop jump from the height of 0.4 m. According to Craig (2004), *neural adaptations* usually occur when athletes respond or react as a result of improved coordination between the CNS signal and proprioceptive feedback. Potteiger et al. (1999) states that *neural adaptations* occur via synchronous firing of the motor neurons or better facilitation of neural impulses to the spinal cord. Obviously, in our research we could not confirm both of these assumptions. It was only possible to indirectly determine whether the *neural adaptations* to increased stretching and amplified workload occurred as the result of plyometric training. However, further research should be carried out for this purpose. For example, research could be directed at determining the effects of plyometric training on muscle activation and performance of lower extremity during jumping exercises. Some researchers carrying out such studies have obtained contradictory results, thus further studies are fully justified (Gehri et al., 1998; Young et al., 1999; Chimera et al., 2004; Miller et al., 2006).

In case of biathletes, jumps are not a specific form of motion. Nevertheless, during training and competition, the biathlete in each stride of the run improves the stretch-shortening cycle of the lower limbs. It is important to notice that competition in Nordic skiing and biathlon is currently held at shorter, sprint distances, what significantly increases stride length, makes the lower limb movement more dynamic, with an increased use of the SSC.

Gymnasts had results which were more persuasive, because both the height of the jump (*h*), and peak power (*P_peak_*) were significantly higher in the DJ (Król and Mynarski, 2010). Sale (1992) presented jump height (*h*) of female gymnasts in the drop jump from different heights. The highest values were observed in a 0.40m height of the drop jump. Presumably, a 0.4m height of the drop jump is the closest to that which gymnasts experience in the landing phase, after performing a handspring or a round off. Komi (Sale, 1992) has provided a similar example with volleyball players, however, their optimal height was 0.6m.

Comparing jump height in the DJ and CMJ, our results do not confirm those of less experienced basketball players presented by Bober et al. (2001). In their research the basketball players jumped from heights ranging from 0.15 m to 0.76 m, and achieved worse results in the DJ than in the CMJ. According to Skurvydas et al. (2002) healthy, untrained men and elite sprinters also achieved a slightly lower jump height during the drop jump than in the counter movement jump. Elite long-distance runners only attained about a 0.05 m greater jump height during the DJ, in comparison to the CMJ.

The drop jump heights which were smaller when compared with the counter movement jump, obtained by Skurvydas et al. (2002) can be explained by the greater load during the DJ that occurs when switching from the eccentric phase of the movement to the take-off phase. This load may be overcome by the elastic energy and stretch reflex, however, adequate strength and coordination are required. If the load is too excessive, it may lead to an inverse myotatic reflex according to Smith and Plowman (2007). It can be assumed that regular long term plyometric training will improve the adaptability of the neuromuscular system. Gehri et al. (1998) proved that both the DJ and the CMJ training group substantially improved their vertical jumping ability after 12 weeks of training. This result was also confirmed by Chimera et al. (2004).

## Conclusions

In normal muscle function, the muscle is stretched before it contracts concentrically. The eccentric-concentric coupling, also referred to as the stretch-shortening cycle, employs the stimulation of the body’s proprioceptors to facilitate an increase muscle fiber recruitment over a minimal time period. Stretch-shortening exercises (*plyometrics*) use the elastic and reactive properties of a muscle to generate maximal force production. The stretch-shortening exercises include various types of jumps, including drop (depth) jumps. Such activity requires muscles, in this case those of the lower limbs, to rapidly and forcefully contract while lengthening (eccentric) at the time of landing. Subsequent rapid and forceful contraction (concentrically) is needed to perform a subsequent jump. Upon landing, the force of the body mass is amplified. The amount of amplification depends on the height of the initial drop. There is agreement about the benefits of basic stretch-shortening principles, but controversies exists in regards to the optimal training routine.

Based on the results obtained from in this study, the following conclusions were drawn:

- Jump heights in different forms of the vertical jump were not significant in elite biathletes. Most likely jumping ability is not the most important factor for success in this sport discipline.
- Drop jumps proved to be a more effective way to increase power output and jumping performance than other vertical plyometric exercises such as the counter movement jump.
- Higher values of jump height and mean power registered in the drop jumps, in comparison to the counter movement jump, indicate a better adaptation of the neuromuscular system of biathletes to increased stretching and a greater workload. The greatest workload was reached by drop jumps from an elevation of 0.4 m. On the basis of this research, it may be concluded that the stretch-shortening cycle is an effective manner for developing strength-speed abilities of biathletes.

## Figures and Tables

**Table 1:** Characteristics of the biathletes

Sex	Age [years]		Body mass [kg]		Body height [cm]		Training experience [years]	

	Mean	SD	Mean	SD	Mean	SD	Mean	SD
Female	18.3	1.32	64.1	4.34	169	5.95	4.8	1.83
Male	19.2	0.70	72.0	4.57	177	3.72	5.5	2.88

**Table 2:** The height of the jump (h), mean power (P_mean_) and peak power (P_peak_) in three types of vertical jumps: the counter movement jump (CMJ), a special vertical jump (touching a bar with the head; SCMJ) and the drop jump (DJ) of female biathletes

Variable		h [m]	P_max_ [W]	P_mean_ [W]
CMJ	Mean	0.374	1821	1028
	SD	0.053	242	138
SCMJ	Mean	0.381	1874	1083
	SD	0.049	233	155
DJ	Mean	0.387	1891	1208
	SD	0.053	235	173

**Table 3:** The height of the jump (h), mean power (P_mean_) and peak power (P_peak_) in three types of vertical jumps: the counter movement jump (CMJ), a special vertical jump (touching a bar with the head; SCMJ) and the drop jump (DJ) of male biathletes

Variable		h [m]	P_max_ [W]	P_mean_ [W]
CMJ	Mean	0.255	1100	678
	SD	0.030	201	134
SCMJ	Mean	0.262	1129	717
	SD	0.031	181	127
DJ	Mean	0.273	1380	916
	SD	0.042	551	366

**Table 4:** A summary of various studies done on the height of the jump (h_CMJ_)

Kollias et al. (2004)	h*_CMJ_***[m]**	Król (2007)	h*_CMJ_***[m]**	Król (2008)	h*_CMJ_***[m]**	Król and Mynarski (2012)	h*_CMJ_***[m]**
**Track and field** n=37		**Ski jumpers** n = 7		**Soccer** n = 134		**Biathlon – men** n = 8	
Age 21.4±3.4y Body mass 73.4±5.8kg Body height 1.82±.07m	**0.364±.047**	Age 19.7±4.2y Body mass 57.1±5.8kg Body height 1.72±.05m	**0.472±.068**	**Goalkeepers** n = 7 Age 25.6±5.3y Body mass 80.5±8.6kg Body height 1.86±.07m	**0.411±.046**	Age 19.2±0.07y Body mass 72.0±4.6kg Body height 1.77±.04m	**0.374±.052**
**Soccer** n = 18				**Soccer Defenders** n =25			
Age 24.6±4.2y Body mass 79.1±7.1kg Body height 1.83±.06m	**0.301±.027**			Age 25.8±4.49y Body mass 82.8±6.1kg Body height 1.85±.04m	**0.398±.046**		
**Volleyball** n = 24				**Soccer Midfielders** n = 71			
Age 24.5±4.2y Body mass 86.5±7.1kg Body height 1.91±.04m	**0.350±.049**			Age 25.6±4.61y Body mass 75.6±6.8kg Body height 1.78±.05m	**0.389±.032**		
**Handball** n = 29				**Soccer Strikers** n = 31			
Age 20.0±1.2y Body mass 83.6±10.0kg Body height 1.87±.08m	**0.286±.043**			Age 24.2±3.96y Body mass 75.9±5.72kg Body height 1.78±.06m	**0.436±.044**		
**Basketball** n = 20							
Age 22.8±3.7y Body mass 92.8±13.2kg Body height 1.94±.10m	**0.300±.045**						
**Rowin**g n = 10							
Age 21.3±2.8y Body mass 80.3±11.0kg Body height 1.84±.05m	**0.306±.057**						

**Table 5:** The results of the Wilcoxon Matched Pairs Ranks test for the height of the jump (h), the mean power (P_mean_) and the peak power (P_peak_), in three kinds of vertical jumps: counter movement jump (CMJ), special vertical jump (touching a bar with the head; SCMJ) and drop jump (DJ) of female biathletes

Parameter	Pair of variables	Number of cases	*T*	*P*
*h*	CMJ ; DJ	8	4.0	**0.0499**
*h*	SCMJ ; DJ	8	8.0	0.1614
*P_mean_*	CMJ ; DJ	8	6.0	0.0929
*P_mean_*	SCMJ ; DJ	8	5.0	0.0687
*P_peak_*	CMJ ; DJ	8	9.0	0.2076
*P_peak_*	SCMJ ; DJ	8	10.0	0.2626

T – value of the Wilcoxon test for the group of N < 25. Bold font refers to a statistically significant value (p < 0.05).

**Table 6:** The results of the Wilcoxon Matched Pairs Ranks test for the height of the jump (h), the mean power (P_mean_) and the peak power (P_peak_), in three kinds of vertical jumps: counter movement jump (CMJ), special vertical jump (touching a bar with the head; SCMJ) and drop jump (DJ) of male biathletes

Parameter	Pair of variables	Number of cases	*T*	*P*
*h*	CMJ ; DJ	8	9.0	0.2076
*h*	SCMJ ; DJ	8	15.0	0.6744
*P_mean_*	CMJ ; DJ	8	A 4.0	**0.0499**
*P_mean_*	SCMJ ; DJ	8	9.0	0.2076
*P_peak_*	CMJ ; DJ	8	14.0	0.5754
*P_peak_*	SCMJ ; DJ	8	18.0	1.0000

**Table 7:** Relationships among performance indices [(h_DJ_ − h_CMJ_), (P_mean DJ_ − P_mean CMJ_), (P_peak DJ_ − P_peakCMJ_)] in the counter movement jump (CMJ), drop jump (DJ) and years of sport experience (YoSP)

Performance indices	R Spearman	p
(*h_DJ_***−***h_CMJ_*) and YoSE	−0.027	0.8110
(*P_mean DJ_ − P_mean CMJ_*) and YoSE	0.264	**0.0190**
(*P_max DJ_ − P_max CMJ_*) and YoSE	0.358	**0.0010**

Bold font refers to a statistically significant value (p < 0.05). *(h_DJ_ − h_CMJ_) – difference between height of the jump in the DJ and the CMJ, (P_mean__DJ_***−***P_meanCMJ_) – difference between the mean power during the take-off phase in the DJ and the CMJ, (P_peak__DJ_***−***P_peak CMJ_) – difference between the peak power during the take-off phase in the DJ and the CMJ.*
